# In the unloaded lower leg, vibration extrudes venous blood out of the calf muscles probably by direct acceleration and without arterial vasodilation

**DOI:** 10.1007/s00421-014-2834-9

**Published:** 2014-02-07

**Authors:** Jochen Zange, Sven Molitor, Agnes Illbruck, Klaus Müller, Eckhard Schönau, Matthias Kohl-Bareis, Jörn Rittweger

**Affiliations:** 1 Institute of Aerospace Medicine, Deutsches Zentrum für Luft- und Raumfahrt, DLR e.V., Cologne, Germany; 2 Medical Faculty, University of Cologne, Cologne, Germany; 3 University of Applied Sciences Koblenz, Rhein Ahr Campus Remagen, Remagen, Germany; 4 University of Cologne, University Children Hospital, Cologne, Germany

**Keywords:** Near infrared spectroscopy, Microcirculation, Whole body vibration, Tonic vibration reflex, Vasomotor control

## Abstract

**Purpose:**

During vibration of the whole unloaded lower leg, effects on capillary blood content and blood oxygenation were measured in the calf muscle. The hypotheses predicted extrusion of venous blood by a tonic reflex contraction and that reactive hyperaemia could be observed after vibration.

**Methods:**

Twelve male subjects sat in front of a vibration platform with their right foot affixed to the platform. In four intervals of 3-min duration vibration was applied with a peak-to-peak displacement of 5 mm at frequencies 15 or 25 Hz, and two foot positions, respectively. Near infrared spectroscopy was used for measuring haemoglobin oxygen saturation (SmO_2_) and the concentration of total haemoglobin (tHb) in the medial gastrocnemius muscle.

**Results:**

Within 30 s of vibration SmO_2_ increased from 55 ± 1 to 66 ± 1 % (mean ± SE). Within 1.5 min afterwards SmO_2_ decreased to a steady state (62 ± 1 %). During the following 3 min of recovery SmO_2_ slowly decreased back to base line. THb decreased within the first 30 s of vibration, remained almost constant until the end of vibration, and slowly recovered to baseline afterwards. No significant differences were found for the two vibration frequencies and the two foot positions.

**Conclusions:**

The relaxed and unloaded calf muscles did not respond to vibration with a remarkable reflex contraction. The acceleration by vibration apparently ejected capillary venous blood from the muscle. Subsequent recovery did not match with a reactive hyperaemia indicating that the mere mechanical stress did not cause vasodilation.

## Introduction

The physiological effects of whole body vibration (WBV) training have been the subject of several studies during the last decade (Rittweger 2010). WBV has always been applied in combination with resistive static or dynamic exercise, which was at minimum weight-bearing during upright standing. The effects of the mechanical stress and the complex afferent input by mere WBV without additional voluntary contraction are not known so far (Rittweger 2010).

When vibration is added to an isometric or dynamic muscle load, the leg muscles react with an increase in EMG activity (Cardinale and Lim 2003; Ritzmann et al. 2013) and an increase in energy turnover (Rittweger et al. 2001; Zange et al. 2009). Moreover, during vibration muscle converts part of the absorbed kinetic energy into heat (Cochrane et al. 2008). All this entails the need for an appropriate blood supply. Kerschan-Schindl et al. (2001) were the first to find a significant increase in blood flow in the popliteal arteria after 3-min standing in a shallow squatting posture on a side alternating vibration platform vibrating at a frequency of 26 Hz. In their recent review, Fuller et al. (2013) summarised eight similar studies on the post-exercise-reactive hyperaemia by WBV and showed a positive correlation between the increase in blood flow and the vibration load defined as the maximum velocity during vibration movement. Near infrared spectroscopy (NIRS) was used to measure the content and oxygenation of haemoglobin in m. vastus lateralis during squatting exercise with and without WBV (Yamada et al. 2005). This study could show a steeper decrease in oxygenation of muscle haemoglobin during exercise with WBV, where post exercise was followed by a transient greater oxygenation than baseline. Muscle hypoxia is the major stimulus for the gradual arterial vasodilation by which muscle increases its oxygen supply during exercise (Casey and Joyner 2011; Sarelius and Pohl 2010). However, it is still under discussion whether during exercise mechanical impacts on erythrocytes or the endothelia may also activate the release of metabolites like ATP or NO, respectively, which are involved in the decrease of vascular tone (Casey and Joyner 2011; Sarelius and Pohl 2010). Acceleration of muscle by WBV may cause large local mechanical stresses which could activate such mechanical mechanisms that involved vasocontrol (Rittweger et al. 2010; Rittweger 2010).

The mechanisms which cause the extra, vibration-specific muscle activity during WBV are also not finally solved. Most authors refer to the tonic vibration reflex which was originally described as a mono-synaptic reflex response to vibration locally applied to a tendon or to the belly of a muscle (Burke et al. 1976a, b; Rittweger 2010). Ritzmann et al. (2013) recently showed that the vibration-specific component of the EMG response to WBV was increased when subjects standing on the vibration plate were bearing additional loads.

In this study, we examined the effects of the mere vibration stimulus in the calf muscle in a setup typical for WBV without applying any further resistive muscle loading. We tested the unloaded and not voluntarily contracting gastrocnemius medialis muscle of seated subjects who had one foot affixed on a side alternating WBV platform. This setup was used to measure the haemodynamic responses by NIRS and the EMG response of mere vibration of the whole lower leg like in WBV, however, without any additional load bearing or voluntary contraction of the lower leg muscles. Furthermore, we compared the effects from unloaded WBV with effects of weak (5 % MVC) to moderate (40 % MVC) voluntary isometric contraction and discussed the probability of a reflex contraction in response to WBV.

The hypothesis was that the vibration caused a visible and EMG detectable contraction with increases in muscle pressure and energy turnover. In consequence, we expected an initial decrease in venous blood volume and therefore a decrease in total haemoglobin (tHb) content and an increase in muscle haemoglobin oxygen saturation (SmO_2_) to a maximum followed by a rapid decrease of SmO_2_ by the increased oxygen demand. We predict that this is followed by SmO_2_ reaching a new steady state lower than baseline. A hyperaemia after vibration would be visible by a rapid and overshooting increase in tHb and SmO_2_. Alternatively, if the calf muscle was not contracting in response to vibration, we hypothesised a simultaneous increase in SmO_2_ and tHb indicating a vasodilation during vibration. The responses in SmO_2_ and tHb to an unloaded WBV like stimulus were furthermore compared with time-matched isometric contractions ranging between 5 and 40 % MVC.

## Methods

### Subjects

Originally the study only consisted of the vibration experiment. Twelve male subjects (age 24 ± 2 years, body mass 77 ± 9 kg, height 183 ± 7 cm) took part in this vibration experiment. Subjects were exposed minimum twice to the vibration before valid experiment data were recorded. This was necessary for optimising the fit of the binding and for familiarisation.

After finishing the data analysis of the vibration experiment the contraction experiment was planned. Unfortunately, only three subjects from the first experiment could be recruited for the second experiment. Therefore, a slightly older group of eight male subjects including five new subjects (age 29 ± 6, *P* = 0.10, weight 82 ± 10 kg, height 184 ± 4 cm) performed the contraction experiment. For technical reasons subjects were selected by their foot lengths to range between 25 and 30 cm. For both experiments, we asked the subjects not to perform intensive training during the examination day and the day before. Experiments were performed between 9 a.m. and 6 p.m.

The study was performed and designed in compliance with the Declaration of Helsinki and subjects gave their written informed consent in accordance with the ethics committee of the Ärztekammer Nordrhein, Düsseldorf, Germany.

### The vibration experiment

**Fig. 1:**
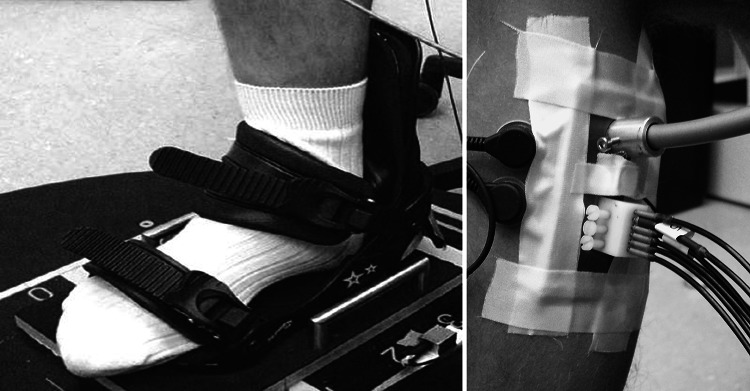
*Left* the right foot of a subject was fixated in a snowboard binding that was attached to a side alternating platform in orthogonal orientation to the rotation axis. *Right* a pair of EMG electrodes and a plate with NIRS optodes was affixed to the belly of the m. gastrocnemius medialis. The upper thick optode is light emitting and the six thin optodes receive light

Results of this study are reported as recommended in a recent consensus publication (Rauch et al. 2010). The subjects sat in front of a side alternating vibration platform (Galileo 900, Novotec Medical GmbH, Pforzheim, Germany) with their right foot affixed to the platform (Fig. 1). Seat height and position were individually adjusted to reach an upright position of the lower leg at a knee angle of 90°. The bare foot was affixed using a small snow board binding which assures vibration transmission to the calf muscles. The foot was either placed parallel to rotation axis of the platform, which during vibration caused an up and down movement of the heel and a small ankle tilt in directions of pronation and supination, or orthogonal to rotation axis to induce an up and down combine with a tilt in directions of plantar flexion and dorsi flexion. In both foot positions, the vibration peak-to-peak displacement was 5 mm at the ankle.

The vibration test included two intervals of 3-min vibration at either 15 or 25 Hz applied in randomised order at randomised foot positions (Table 1). Three min baseline was followed by 3-min vibration, 3-min recovery, 3-min vibration, and 3-min final recovery, respectively.

**Table 1:** Randomised order of test conditions in two groups of subjects

	Test 1		Recovery ≥1 day	Test 2	
	Vibration 1	Vibration 2		Vibration 1	Vibration 2
Group 1 (*n* = 6)	15 Hz orthogonal	25 Hz orthogonal		15 Hz parallel	25 Hz parallel
Group 2 (*n* = 6)	25 Hz parallel	15 Hz parallel		25 Hz orthogonal	15 Hz orthogonal

The time line of both tests comprised 3-min baseline, 3-min vibration 1, 3-min recovery, 3-min vibration 2, and 3-min final recovery, respectively. The foot was placed on the vibration plate orthogonal or parallel to the rotation axis of the plate. The vibration peak-to-peak displacement was 5 mm at the ankle

### The contraction experiment

Foot plantar flexion torque was measured at a 90° foot angle using dynamometer (Biodex System3, Biodex Medical Systems, New York, NY, USA). Subjects sat upright with their legs in horizontal position. Since the examined medial gastrocnemius muscle produces the greatest force with extended knee, this posture was chosen for the contraction experiment. At first maximum voluntary contraction (MVC) was determined as the highest torque reached in sequences of three trials of 5 s duration each separated by 60 s recovery. During the contraction experiment each subject performed four tests at different levels corresponding 5, 10, 20, and 40 % MVC, respectively. The 5 % MVC was chosen as the lowest contraction level that could be reasonably controlled by the subjects. The 40 % MVC level typically represents a contraction under functional ischaemic conditions. The four contractions were arranged in two sets containing a baseline period of 3 min and two contractions of 3 min each followed by 3 min recovery. The two sets were interrupted by break of 60 min duration. One group of four subjects performed the two exercise sets with increasing order of torque, while the other four subjects performed the two sets with decreasing torque.

### Measurement of haemoglobin content and haemoglobin oxygen saturation in the muscle tissue

The concentrations of oxygenated and desoxygenated haemoglobin (HbO_2_, HbH given in μmol/l tissue) in the medial gastrocnemius muscle were measured using NIRS. The NIRS device (Rhein Ahr Campus Remagen, University of Applied Sciences Koblenz, Remagen, Germany) emitted light from a 50 W halogen light source through a fibre bundle (diameter 5 mm) transcutaneous into belly of the muscle. The scattered light emitted from the skin was detected by a linear array of six equidistant optical fibres of 1 mm diameter that were placed at distances between 22.5 and 35.0 mm from the emitting fibre. The whole set of optical fibre bundles (optodes), i.e. the plane ends of the glass fibres, was affixed in a soft plastic plate in almost orthogonal position to the muscle surface. The optode plate was taped over the belly of the medial gastrocnemius muscle with the line of optodes approximately parallel to the muscle fibres. The concentrations of HbH and HbO_2_ were calculated from attenuation spectrum between 650 and 900 nm that was sampled every 2.5 s. Haemoglobin parameters were calculated according to spatial-resolved spectroscopy (Suzuki et al. 1999) and details of the setup can be found in the literature (Geraskin et al. 2009).

Changes in muscle blood content were monitored as changes in total haemoglobin (tHb, μmol/l) calculated as the sum of HbH and HbO_2_. Furthermore, muscle oxygen saturation (SmO_2_ given in %) was calculated as 100 × HbO_2_/tHb. In our study, we examined lean male subjects who typically show an adipose tissue thickness of <5 mm at the position of the gastrocnemius muscle. With our experimental setup, the potential effects of such thin adipose tissue layers on the measured absolute tHb and SmO_2_ values are much smaller than the inter-subject variability and can therefore be neglected (Geraskin et al. 2009).

### EMG recording during the vibration experiment

During the vibration experiment bipolar EMG was recorded from the medial gastrocnemius muscle with a pair of active electrodes attached beside the array of NIRS optodes. EMG was recorded within 10–500 Hz at a sampling rate of 2,000 Hz using a Noraxon Myosystem 1400 (Velamed, Cologne, Germany).

### Statistical analysis

Data are shown as mean values ± standard error (SE). Repeated measure ANOVA was used to test for significant effects of vibration or voluntary contraction and for significant effects due to frequency, the order of applied frequency, and the foot position in the vibration experiment as well as the four different torque levels in the contraction experiment. Tukey’s post hoc test was used where applicable. Significance was accepted at *P* < 0.05 and high significance at *P* < 0.01.

## Results

### Vibration failed to induce a detectable reflex contraction of the calf muscle

At both foot positions and at both vibration frequencies lower leg muscles did not visibly contract in response to vibration. Also the subjects could not sense reflex contractions. The EMG records showed a dominant, rhythmic signal in the frequency of the corresponding vibration frequency. These signal waves were regular but not sinusoidal. After transferring intervals of 1,024 ms (1,024 data points) into the frequency domain using spectral analysis, the vibration frequency and three harmonic frequencies were cut out with a band width of 5 Hz. The remaining very low EMG signal could either be due to off-resonance movement artefacts or a low activity of motor unit action potentials. As there is no means to clarify this, EMG signals were not further analysed.

### Effects of vibration on SmO_2_ and tHb

**Fig. 2:**
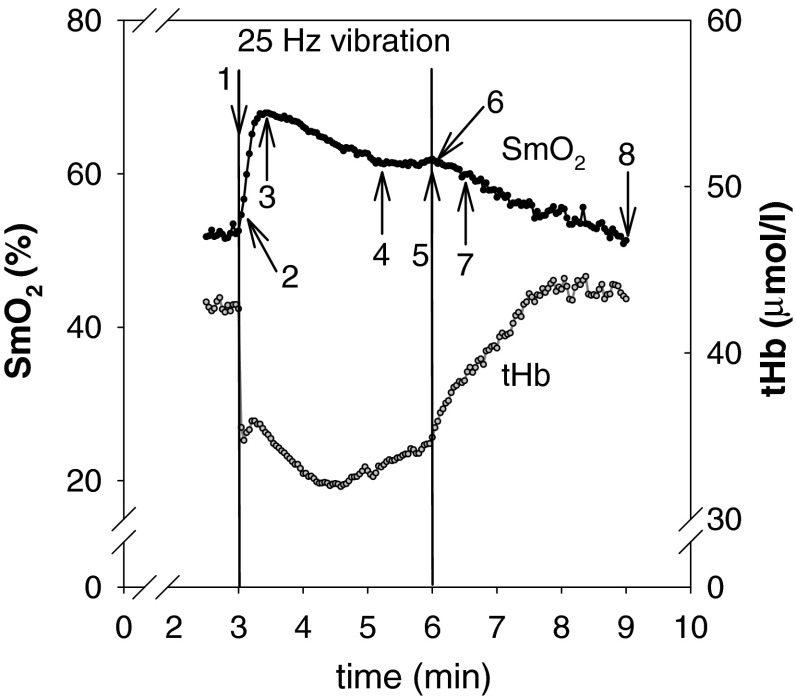
Example of typical records of SmO_2_ and tHb in the medial gastrocnemius muscle during and after vibration. In this example, the vibration frequency was 25 Hz. The record only shows the first vibration stimulus (3–6 min). The foot was placed parallel to the axis of the platform and was therefore lifted without rotation in the ankle. The *numbers* and *arrows* indicate the time points selected for the statistical evaluation of SmO_2_ and tHb, respectively (compare Fig. 3); *1* last data point at rest, *2* 2.5 s vibration, *3* maximum SmO_2_, *4* steady state of SmO_2_, *5* 180 s vibration, *6* 2.5 s recovery, *7* 30 s recovery, *8* 180 s recovery

Typical records of SmO_2_ and tHb during and after 3 min of vibration (25 Hz) are shown in Fig. 2. This time course was observed stereotypically in all experiments, with the onset of vibration always resulting in an increase in SmO_2_ and a rapid drop of tHb. Maximum SmO_2_ was then reached after 30 ± 5 s (mean ± SD) followed by a decline and a steady period which lasted till the end of vibration. After vibration, both SmO_2_ and tHb slowly recovered back to baseline within 3 min. This general pattern of the SmO_2_ and tHb responses to vibration was observed in all subjects. Therefore, characteristic time points in the course of SmO_2_ (see Fig. 1) were selected and the corresponding values for both SmO_2_ and tHb were statistically evaluated. The magnitude of the effects neither depends on the vibration frequency (15, 25 Hz), nor on the addition or absence of an ankle rotation by the two foot positions on the vibration plate, nor on the order of the applied stimuli (all *P* values >0.1, also see Table 2). Since for SmO_2_ and tHb no group effect was significant, data from both vibration frequencies and both foot positions were pooled for further analysis.

**Table 2:** *P* values of group effects from repeated measure ANOVA on the time courses of SmO_2_ and tHb in the medial gastrocnemius muscle during and after the application of vibration

Group	*P* values for SmO_2_	*P* values for tHb
Frequency order	0.713	0.933
Frequency	0.357	0.882
Foot placement	0.105	0.551

Twelve subjects were examined. Significance was accepted for *P* < 0.05. Group effects were the order of the applied vibration frequency (15 Hz first or 25 Hz first), the frequency (15 or 25 Hz), and the placement of the foot on the vibration platform resulting in lifting of the whole foot or a small rotation in the ankle. The selection of time points used in the repeated measure ANOVA is shown in Fig. 1. For both SmO_2_ and tHb all group effects were not significant. Therefore, all groups were pooled for further analysis

**Fig. 3:**
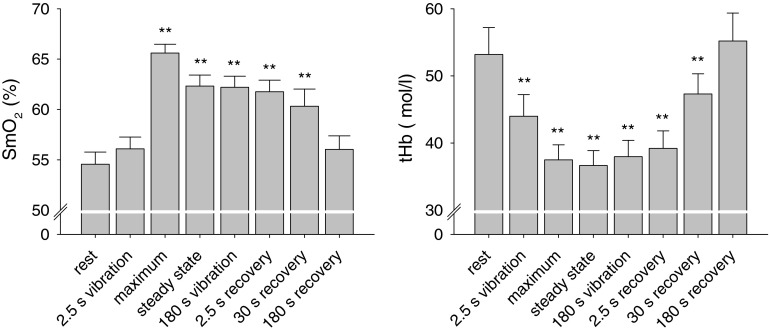
Mean values (±SE, *n* = 12) of SmO_2_ and tHb in the medial gastrocnemius muscle at baseline and selected time points during and after the application of vibration (compare Fig. 2). Data were pooled from experiments using 15 and 25 Hz and two foot positions. Vibration significantly affected both SmO_2_ and tHb (*P* < 0.01, repeated measure ANOVA; **P < 0.01, Tukey’s post hoc test). Both SmO_2_ and tHb returned to baseline after 180 s recovery

On average SmO_2_ increased from 55 ± 1 % to a maximum of 66 ± 1 %. SmO_2_ slowly decreased until it reached a steady state at 62 ± 1 % after approximately 1.5 min of vibration. During recovery mean SmO_2_ linearly decreased back to base line. In parallel tHb decreased from 53 ± 4 μmol/l at baseline to 37 ± 2 μmol/l when SmO_2_ had reached its maximum. This value almost remained on the same level until the termination of the vibration stimulus. During recovery tHb returned to baseline in a slow exponential-like time course (see Fig. 3).

### The effects of isometric contractions on SmO_2_ and tHb

**Fig. 4:**
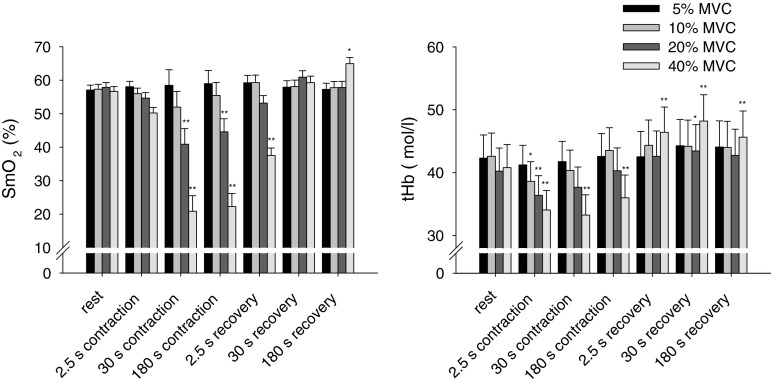
Mean values (±SE, *n* = 8) of SmO_2_ and tHb in the medial gastrocnemius muscle at baseline and selected time points during and after an isometric contraction at levels between 5 and 40 % of MVC. For both SmO_2_ and tHb only at 20 and 40 % MVC repeated measure ANOVA indicated significant effects of contraction with respect to initial rest (*P < 0.05, **P < 0.01, Tukey’s post hoc test)

Initial values of tHb were lower in the contraction experiment than in the vibration experiment. This difference was obviously caused by the different posture of the lower leg due to knee extension. SmO_2_ started at comparable levels in both experiments (compare Figs. 3, 4).

As expected, the effects of isometric contraction on SmO_2_ and tHb depended on the torque of muscle contraction (see Fig. 4). Contractions at 5 % MVC did neither affect SmO_2_ nor tHb. Contractions at 10 % MVC resulted in moderate decreases of SmO_2_ and tHb which, however, did not reach significance. SmO_2_ significantly decreased during 20 % MVC (58 ± 1–39 ± 5 %) and 40 % MVC (57 ± 1–18 ± 5 %). After contraction, SmO_2_ rapidly recovered with 30 s and showed an overshoot in response to the 40 % MVC contraction. THb also decreased during 20 % MVC (40 ± 4–37 ± 3 μmol/l) and 40 % MVC (41 ± 4–33 ± 3 μmol/l). After contraction, tHb recovered until the first point of measurement (2.5 s) and showed an overshoot in response to the both contraction levels.

## Discussion

In the belly region of the voluntarily relaxed gastrocnemius medialis muscle the vibration of the whole lower leg resulted in a rapid decrease in tHb and an increase of SmO_2_ reaching a maximum after 30 s. The rapid decrease in tHb strongly suggests a reduction of the total blood content, and the increase in SmO_2_ indicates that more non-oxygenated than oxygenated blood is extruded. Taken together, this could well be explained by a blood extrusion from the venules. The maximum in SmO_2_ was followed by a transient decrease to a new steady state significantly higher than baseline. After vibration, tHb and SmO_2_ slowly returned back to baseline within 3 min. The pattern of the vibration effect was highly stereotypic between subjects. Time course and magnitude of the vibration effect were neither affected by the two vibration frequencies (15, 25 Hz) nor by mode of vibration induction with small foot tilting in the pronation—supination or plantar flexion—dorsi flexion planes, respectively.

During the first 30 s, the decrease in tHb and the increase in SmO_2_ caused by a WBV-like stimulus on the unloaded gastrocnemius muscle match with the previous findings when WBV was applied on subjects in shallow squat standing position (Rittweger et al. 2010). However, in standing position these initial effects of WBV were smaller than in the unloaded muscle. The later changes in tHb and SmO_2_ during vibration observed in standing subjects were very variable and probably caused by irregular calf muscle contractions to stabilise stance during WBV. In so far the effects of WBV in standing posture differ to a large extent from the stereotypic time courses of WBV effects in completely unloaded muscles in our study. Even bigger are the differences between the effects of WBV on the unloaded calf muscle observed here and the effects of WBV on thigh muscles during resistive squatting exercise which was causing a steeper decrease in SmO_2_ indicating a greater oxygen extraction than during mere squatting exercise (Yamada et al. 2005).

Principally, driving forces for the initial extrusion of venous blood by the WBV-like stimulus on the unloaded calf muscle could result from extravascular pressure by a reflex muscle contraction or by forces induced by passive motions between muscle and vessels. Furthermore, vibration may accelerate blood itself. It is well established that WBV added to resistive exercise causes an excess EMG activation (Cardinale and Lim 2003; Ritzmann et al. 2013) and energy turnover (Rittweger et al. 2001; Zange et al. 2009). However, the WBV-like vibration of the unloaded lower leg failed to induce a remarkable reflex reaction in the calf muscle. Such a contraction was neither visible to the experimenter nor sensed by the subjects. In accordance with the previous findings (Abercromby et al. 2007), the EMG record showed rhythmic artefacts in the corresponding vibration frequency and its harmonics. In our experiments on voluntary relaxed muscles this artefact signal was so dominant that any further analysis of the EMG signal after filtering was not feasible for technical reasons. In our experiment the foot was placed in two directions on the platform where the whole lower leg was vibrated either by lifting the leg or by causing a small tilt in the ankle. We expected that a potential reflex contraction of the gastrocnemius would be more likely caused when the ankle was tilted. Furthermore a vibration reflex contraction should be stronger at 25 Hz than at 15 Hz. However, the extrusion of venous blood from the muscle was independent from both the foot position and the frequency which gave further evidence that the initial blood extrusion by vibration was not caused by a reflex contraction.

Nevertheless, in our experiment the observation of a very weak muscle contraction by the vibration stimulus could be hampered by the motion of the leg, the very strong sensation of vibration by the subjects, or the EMG artefact. Therefore, we compared the effects of vibration on tHb and SmO_2_ with the corresponding effects of voluntary isometric contraction. The lowest contraction level that could be controlled by the subjects was 5 % MVC. This very low contraction level did not cause any mean reactions of tHb and SmO_2_. The higher force levels of 10, 20, and 40 % MVC resulted in the expected pattern of a decrease in tHb and decrease in SmO_2_ caused by a functional muscle ischaemia and increasing oxygen consumption both increasing in significance with increasing force levels. The reaction pattern to vibration, i.e. an increase in SmO_2_ followed by a decrease to a steady state higher than baseline, respectively, was not observed at any tested level of voluntary contraction.

Taking the evidence together, namely (1) the lack of any visible, sensible or EMG-based muscle activity, (2) the lack of any different effects of 15 and 25 Hz and any effect of ankle-tilt involvement, and (3) the different effects of voluntary isometric contraction on tHb and SmO_2_ during and after contraction we propose that the clear-cut responses of tHb and SmO_2_ to vibration on the unloaded lower leg were independent from muscle contraction. We therefore propose a direct mechanical action of vibration accelerating partially deoxygenated blood out of the small and NIRS visible venules into larger veins totally absorbing near infrared light. The unknown mechanisms which transfer the oscillating acceleration into a directed force probably utilise the direction of blood flow and an involvement of venous valves.

Recoveries of tHb and SmO_2_ after vibration were very slow and did not follow the pattern of a reactive hyperaemia that was observed, e.g. after an isometric contraction at 40 % MVC (see Fig. 3). When applied to a relaxed muscle, vibration did not result in dilatation of resistive arteries. During muscle exercise the control of blood flow by the vascular tone is generally determined by metabolic factors which predominantly depend on the balance of oxygen supply and the oxygen demand of the working muscle (Casey and Joyner 2011). Besides this, also minor involvement of mechanical stimuli on the mechanisms controlling arterial vasodilatation in muscle is discussed like the ATP release from red blood cells by deformation or the NO release from endothelia cells by shear stress (Sarelius and Pohl 2010). WBV is a strong mechanical stimulus for the whole muscle tissue that force the muscle to deal with kinetic energy which is determined by both frequency and amplitude (Rittweger 2010). Our results show that mechanical forces, at least when applied by vibration, did not induce vasodilation of resistive arteries in the m. gastrocnemius medialis. Moreover, during vibration the transient decrease of SmO_2_ after the maximum and the parallel small increase in tHb could be interpreted as a moderate vasoconstriction as an autonomic response of the subjects to the uncomfortable sensation of the vibration of the lower leg. Such a vasoconstriction would have forced a higher oxygen extraction from the blood that would match with the observation of the decrease of SmO_2_ to a new steady state in our experiments.

The absence of a vasodilation in the unloaded calf muscle stimulated by a WBV like stimulus seems to be in contrast with the frequency-depending reactive hyperaemia that was found in response to the combination of WBV with resistive exercise (Fuller et al. 2013; Kerschan-Schindl et al. 2001). As previously mentioned, in the combination of WBV and resistive exercise, WBV additionally activates the muscle (Cardinale and Lim 2003; Ritzmann et al. 2013), increases muscle energy turnover (Rittweger et al. 2002; Zange et al. 2009), causes a rise in muscle temperature by the conversion of vibration energy into heat (Cochrane et al. 2008), and causes a steeper muscle deoxygenation (Yamada et al. 2005). Since WBV applied to the unloaded muscle did not cause a vasodilation when WBV seems to cause an exclusive mechanical stimulus, we conclude that WBV added to resistive working muscle also does not act via mechanically triggered pathways. Instead, WBV added to resistive exercise likely induces vasodilation exclusively by factors related to the enhanced muscle hypoxia or heating caused by this combination of exercise modes (Cochrane et al. 2008; Yamada et al. 2005).

## Conclusion

In conclusion, our study on the medial gastrocnemius muscles showed that vibration applied to the unloaded, relaxed lower leg induces alterations in the content of oxygenated and deoxygenated blood in the belly region of the muscle that could clearly be interpreted as an extrusion of partially desoxygenated blood from the small veins. We could not find any evidence for a reflex contraction in response to the WBV in the unloaded muscle. We therefore propose a direct vibration-depending mechanism for the initial extrusion of blood from the muscle. Furthermore, vibration when separated from any further voluntary active or resistive muscle contraction did not result in post-vibration hyperaemia by vasodilation. Since such a strong and permanent mechanical stimulus applied in the absence of hypoxia did not reduce vascular tone in the gastrocnemius muscle, one could conclude that generally in this muscle mechanical stimuli seemingly do not activate signal pathways controlling vasodilation in response to exercise.

## Abbreviations

EMG: Electromyography
MVC: Maximum voluntary contraction
NIRS: Near infrared spectroscopy
SmO_2_: Muscle haemoglobin oxygen saturation
tHb: Total haemogloin
WBV: Whole body vibration
